# Gamma irradiation effects on photoluminescence and semiconducting properties of non-conventional heavy metal binary PbO–Bi_2_O_3_ glasses

**DOI:** 10.1038/s41598-024-72110-x

**Published:** 2024-09-25

**Authors:** M. A. Marzouk, I. S. Ali

**Affiliations:** 1 Glass Research Department, National Research Centre, 33 EL Bohouth St. (Former EL Tahrir St.), Dokki, P.O.12622, Giza, Egypt; 2 Pharos University in Alexandria, Canal Mahmoudiah Street, Smouha, Alexandria Egypt; 3 Higher Institute of Optics Technology, Heliopolis, Cairo, Egypt

**Keywords:** Optical, Heavy metal, Glass, Gamma irradiation, Glasses, Glasses

## Abstract

Non-conventional heavy metal oxide glasses have attracted great interest owing to their unique optical properties and their radiation shielding behavior. Non-conventional glasses of main chemical composition (100 − x) PbO–xBi_2_O_3_ where x = 35, 30, 25, 20, 15, 10, and 5 were prepared through the conventional melting and annealing approach. X-ray diffraction measurements denoted the amorphous nature of the prepared glasses. The optical absorption in the UV–visible range recorded strong UV-near visible absorption spectra that correlated to trivalent Bi^3+^ ions. The optical band gap E_opt_, Urbach energy ∆E, and the refractive index were identified for the prepared glasses employing the cognizant theories. The variations in the optical parameters have been associated with the increasing Bi_2_O_3_ and the doses of γ- irradiation. The photoluminescent properties of the prepared non-conventional binary Bi_2_O_3_–PbO glasses were recorded in the visible range after UV excitation and the color coordinates are located and distributed in the hue violet degree. FT-IR spectroscopic measurements before and after gamma irradiation were applied to investigate the structural changes in the binary heavy metal PbO–Bi_2_O_3_ glasses. FTIR data specified that the glass network is composed of different structural building units from BiO_3_/BiO_6_ and PbO_3_/PbO_4_ depending on the addition ratio between PbO and Bi_2_O_3_.

## Introduction

Glass is regarded as an amorphous solid material that can be obtained via the supercooling of melts slowly to prevent crystallization. Generally, the chemical composition of most types of glass is based on glass-forming oxides that are characterized by their low rate of crystallization like SiO_2_, P_2_O_5_, B_2_O_3_, and GeO_2_^1,2^.

One of the key factors in the formation of glass is the critical cooling rate of the glass melt. The critical rate of cooling in glass formation refers to the minimum cooling rate necessary to prevent crystallization and enable a liquid to transition into a glassy state^1,2^. This concept is vital in understanding the glass-forming ability of different materials. Melts that can be cooled to form glasses without requiring extremely high cooling rates are classified as good glass formers, indicating that they possess favorable characteristics for glass formation. Conversely, melts that cannot achieve glass formation without resorting to extreme cooling rates are deemed non-glass formers. A key factor influencing the glass-forming ability of a particular melt is the temperature dependence of its viscosity. As the temperature decreases, the viscosity of the liquid increases, affecting the material’s ability to transition into a glassy state. Thus, the interplay between cooling rates, crystallization tendencies, and viscosity plays an effective role in determining whether a material can successfully form a glass.

Other special types of non-traditional glasses with non-forming oxides have been developed for advanced applications. For instance, chalcogenide glasses are used in electro-optical devices such as memory panels, halide glass substituted silica fibers in the communication field, ionic conducting glasses used in the manufacture of batteries, and metallic glasses have extended applications in optical, electrical, and magnetic purposes^3,4^.

Conversely, Non-traditional oxide glasses represent a novel category of glasses that do not adhere to Zachariasen’s rules for glass formation. These rules specify the general requirements for creating oxide glasses, including that no oxygen atom should be bonded to more than two cations, the cation coordination number should be either 3 or 4, oxygen polyhedra should share corners rather than edges or faces, and at least three corners must be shared. Such types of glasses are primarily characterized by the absence of conventional glass network formers^5^.

Heavy metal non-conventional glasses have attracted much interest because their glass building network formed from non-conventional glass formers leads to many interesting physical and chemical properties^6–12^.

Numerous studies^13–15^ have demonstrated that bismuth, despite being classified as a non-conventional glass former with a field strength of less than 1.3, can effectively substitute traditional glass formers like boron, in borate-based glasses. This substitution is particularly noteworthy because bismuth is capable of forming stable glasses across a wide compositional range, largely due to its hyper-polarizability. The unique properties of Bi^3+^ ions, characterized by their high polarizability and low field strength, contribute to an increased concentration of non-bridging oxygen in the glass matrix compared to glasses made with more common glass formers^13–15^. This phenomenon highlights the versatility of heavy metal oxide glasses that incorporate bismuth, which has gained considerable attention in recent years. Bismuth serves a dual function in these glasses: at higher concentrations, it acts as a glass network former, while at lower concentrations, it functions as a modifier. This duality enhances the structural and functional properties of the glass, making it an attractive material for various applications. Additionally, Bi^3^⁺ ions exhibit remarkable luminescence characteristics across a wide range of the electromagnetic spectrum, including the high-energy ultraviolet–visible (UV–Vis) region and the low-energy infrared (IR) region. This luminescent behavior further underscores the potential of bismuth-based glasses in advanced technological applications^13–15^.

In previous studies^16–19^, heavy metal oxide HMO glasses were defined as glasses that are containing over 50 weight % percent of heavy metal bismuth and/or leadership that contribute to the formation of glass network and are considered the main former. These glasses are characterized by their high density, high polarizability, and high nonlinear optical behavior. The unique properties of PbO or Bi_2_O_3_ glasses are established from the dual role of these oxides as glass formers or modifiers^13–15,20^. The non-conventional glasses are known to be glasses that do not contain any conventional glass former such as SiO_2_, B_2_O_3_, P_2_O_5_, GeO_2_, etc. with long infrared cut-off and possess a high third-order non-linear optical susceptibility^13–15,20^.

Although bismuth oxide or lead oxide are not considered glass formers when mixed in a particular portion they can form a special type of glass and in such a state, they are nominated as non-conventional heavy metal oxides as described by Pand and Ghosh^21^. Depending on the concentration, higher content of HMO allows the introduction of the glass network structure with the formation of the glass network bridging oxygens BO and it is referred to as a network former, while other levels of concentration for instance lower content can allow HMO to breakdown the BO to form new non-bridging oxygens NBO and HMO indicated as a glass modifier as reported by Pan and Ghosh^21^.

The present study aimed at preparing a new class of glasses based on heavy metal oxides at different ratios between PbO and Bi_2_O_3_ using the conventional melt and annealing method. It was also necessary to test the shielding behavior of the formed glasses toward gamma irradiation to estimate their radiation shielding ability.

The use of glasses composed of heavy metal oxides, particularly PbO and Bi_2_O_3_, shows significant potential in radiation shielding applications for several compelling reasons. Firstly, both lead oxide (PbO) and bismuth oxide (Bi_2_O_3_) possess high atomic numbers that effectively attenuate gamma rays, a critical requirement for materials designed for radiation shielding. Secondly, adjusting the PbO and Bi_2_O_3_ ratios allows us to customize the glass composition, optimizing its ability to shield against gamma irradiation to meet specific protection needs. This versatility makes these glasses suitable for a wide range of applications, from medical facilities to nuclear industries. Moreover, incorporating heavy metal oxides into glass formulations ensures compliance with rigorous environmental and safety standards governing radiation shielding materials, particularly crucial in industries prioritizing radiation protection. Lastly, the commercial viability of these glasses is underscored by increasing demand across sectors such as healthcare and nuclear energy. Their adaptability for applications like shielding windows and specialized containers further enhances their appeal, supported by ongoing research and development fostering innovation and facilitating their transition from laboratory concepts to market-ready solutions.

In the current study, we focused our aid on the preparation of heavy metal lead glasses with varied content of bismuth oxide as a binary low melt heavy metal glass then a characterization of the building structure with the FTIR technique. Also, it was necessary to evaluate the optoelectronic behavior of these glasses and their optical band gap together with photoluminescence properties to detect their validity of application as a semiconducting glass. All measurements were carried out on the prepared glasses before and after irradiation with different doses of gamma irradiation to show the shielding behavior of the prepared HMOG.

## Experimental details

The glass with the chemical composition listed in Table 1 was prepared via a conventional melt-quenching technique. Appropriate amounts of pure powders of reagents Pb_3_O_4_ and Bi_2_O_3_ (from Sigma-Aldrich Company, purity 99.9%) were weighed and mixed then melted in alumina crucibles at 1050 °C for 15 min in an electrical furnace. The formed melt was gently stirred to reach homogeneity then complete melting was carried out after 10 min at 1050 °C. The melt of each batch was poured at room temperature between two molds of stainless steel and transferred to the annealing furnace that regulated at a maximum temperature of 300 °C to prevent and reduce any probability of thermal shock. The annealing muffle was switched off and left to cool to room temperature.

**Table 1:** Chemical composition, optical band gaps (E_opt_), Urbach energy (∆E) and refractive index (n) of the studied glasses.

Sample	Oxide %		E_op_/eV			∆E/eV			n		
	PbO	Bi_2_O_3_	0 Mrad	2 Mrad	10 Mrad	0 Mrad	2 Mrad	10 Mrad	0 Mrad	2 Mrad	10 Mrad
S1	65	35	2.377	2.276	2.355	0.526	0.720	0.863	2.608	2.638	2.622
S2	70	30	2.284	2.255	2.269	0.620	0.773	0.774	2.652	2.646	2.660
S3	75	25	2.299	2.272	2.282	0.685	0.784	0.764	2.645	2.651	2.646
S4	80	20	2.324	2.288	2.321	0.471	0.758	0.729	2.632	2.634	2.634
S5	85	15	2.303	2.281	2.304	0.521	0.858	0.759	2.626	2.659	2.652
S6	90	10	2.257	2.249	2.278	0.511	0.678	0.749	2.630	2.664	2.649
S7	95	5	2.292	2.264	2.261	0.639	0.687	0.763	2.641	2.666	2.664

Table 2 presents the results of an X-ray fluorescence (XRF) analysis carried out on a subset of samples taken from the prepared glasses. The purpose of this analysis was to ascertain the final chemical composition of the glass samples. XRF was used to obtain detailed information about the elemental constituents of the glasses, which is essential for understanding their characteristics and potential uses.

**Table 2:** XRF analysis of some selected samples from the prepared glasses.

Sample	Oxide %							
	PbO	Bi_2_O_3_	Fe_2_O_3_	CaO	K_2_O	MgO	Mn	ZnO
S2	69.73	30.01	0.005	0.01	0.03	0.01	0.001	0.005
S6	89.87	9.96	0.007	0.03	0.03	0.01	0.005	0.00

X-ray diffractograms of the prepared binary heavy metal non-conventional glasses were recorded using a Bruker AXS diffractometer CD8-ADVANCE with Cu-Ka radiation, operating at 40 kV and 10 mA. The diffraction data were recorded for 2θ values between 4 and 70° and the scanning rate was 10°/min.

The optical UV–visible absorption spectra in the range 200–1100 of well-polished surfaces glassy samples with a thickness equal to 2 mm ± 0.1 mm were measured at room temperature using a computerized recording spectrophotometer type T80t, PG Instrument Ltd., England.

The bandgaps were calculated from the optical absorption coefficient using the formula by Mott and Davis^22^.

1 $$\alpha hv = B(hv - E_{opt} )^{n}$$

where; α is the absorption coefficient, *h* is Planck’s constant, *ν* is the frequency of the incident light, *E*_*opt*_ is the optical bandgap energy, *B* is a constant that depends on the material, and *n* is a parameter that indicates the nature of the transition (for example, n = 1/2 for direct transitions and *n* = 2 for indirect transitions). In this context, the optical absorption coefficient (*α*) is related to the energy of the incident photons, allowing to plot (α*hν*)^1/n^ against *hν* to determine *E*_*opt*_ from the intercept of the linear portion of the plot with the energy axis.

The absorption spectra of glass samples are significantly influenced by their thickness, a relationship that is well explained by Beer’s law. This law states that the absorbance of a material is directly proportional to its concentration and the path length through which light travels. To accurately assess the absorption characteristics of our glass samples without the confounding variable of thickness, we undertook a systematic calculation of the absorption coefficient for each sample. By determining the absorption coefficient (α), we aimed to isolate the intrinsic absorption properties of the materials, thereby allowing for a clearer understanding of their optical behavior. The methodology employed for calculating the absorption coefficient involved several precise steps, which we detail in the following Eq.^23^:

2 $$\alpha = \frac{2.303 \times A}{t}$$

where (A) denotes the measured absorbance, and t represents the thickness of each sample in centimeters.

The Urbach energy (ΔE) was estimated from Urbach’s mathematical relationship (3)^24^:

3 $$\alpha (\nu ) = B\exp \left( {\frac{hv}{{\Delta E}}} \right)$$

The ΔE values can be estimated by plotting drawn between lnα and photon of energy *hv* and taking the slope at the linear region of the curves.

The values of the refractive index were estimated using Dimitrov and Sakka^25^ model as follows;

4 $$\frac{{n^{2} - 1}}{{n^{2} + 1}} = 1 - \sqrt {\frac{{E_{opt} }}{20}}$$

The photoluminescence spectral measurements were obtained under UV excitation using a fluorescence spectrometer Type Jasco FP-6500, Japan, equipped with a xenon arclamp at the excitation light source. The scan speed is 0.1 s step-1 with a step length of 0.25 nm and a slit width of 0.2 nm. The CIE chromaticity coordinates of the prepared material were estimated according to the CIE1931 chromaticity diagram. The CIE1931 chromaticity diagram was applied to identify the emitted color from light sources using three dimensionless quantities x̄(λ), ȳ(λ), and z̄ (λ). The tristimulus values for a non-monochromatic light source with spectral relative power P(λ) are given by:

5 $$X = \smallint P\left( \lambda \right)\overline{x}\left( \lambda \right)d\lambda$$

6 $${\text{Y}} = \smallint {\text{P}}\left( \lambda \right)\overline{y}\left( \lambda \right){\text{d}}\lambda$$

7 $${\text{Z}} = \smallint {\text{P}}\left( \lambda \right){\overline{\text{z}}}\left( \lambda \right){\text{d}}\lambda$$

where X, Y, and Z are the tristimulus values that identify the three primary light colors red, green, and blue that needed to match the color P(λ). and from the tristimulus values the color chromaticity coordinates x and y can be determined using the following expression^26,27^;

8 $$\text{x }= \frac{\text{X}}{\text{X}+\text{Y}+\text{Z}}$$

9 $$\text{y }= \frac{\text{Y}}{\text{X}+\text{Y}+\text{Z}}$$

10 $$\text{z }= \frac{\text{Z}}{\text{X}+\text{Y}+\text{Z}}$$

Infrared absorption spectra of the glasses were recorded within the range 400–4000 cm^−1^using FTIR spectrometer Type Mattson 5000, USA at room temperature before and after gamma irradiation. the KBr disc technique was applied for FTIR measurements.

The applied gamma irradiation source was ^6**0**^Co gamma cell 2000Ci, INDIAN, with a dose rate of 1.5 rad/s at a temperature of 30 °C and with a final collective dose of 2 and 10 Mrad.

## Results and discussions

### Amorphous nature of the prepared glasses

**Fig. 1:**
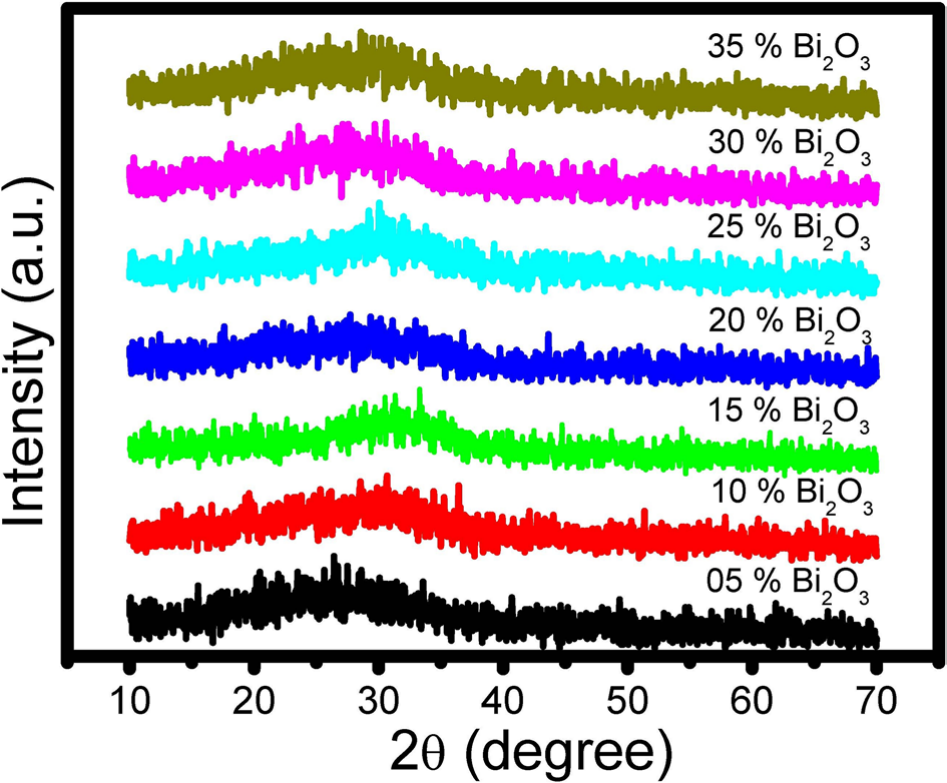
X-ray diffraction of unconventional binary PbO–Bi_2_O_3_ glasses.

The synthesis of glasses with non-conventional glass formers is considered the main objective in the current study. Hence, x-ray diffraction measurements were applied to prove the amorphous building structure of the prepared glasses. Figure 1 depicts the x-ray diffraction of the prepared non-conventional binary PbO–Bi_2_O_3_ glasses. The XRD measurements indicate no characteristic or distinguished peaks for any crystals precipitated during melting or after pouring glasses. According to XRD results, the amorphous nature is allowed for parent binary lead–bismuth glasses within the composition range (60 + x)PbO–(40 − x)Bi_2_O_3_, whereas x = 5, 10, 15, 20, 25, 30, 35 and matched with the rules of glass random network nature as explained by glass-forming theories^2^.

### Optical UV–visible absorption spectral measurements

**Fig. 2:**
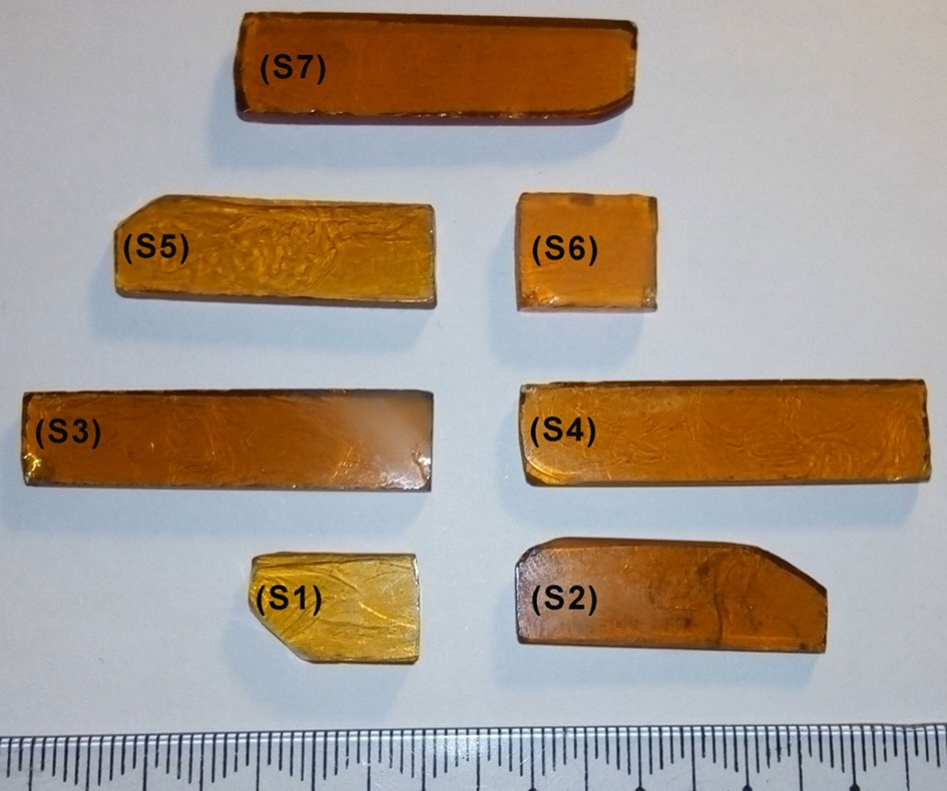
Photograph of the prepared unconventional binary PbO–Bi_2_O_3_ glasses.

The next relative evaluation step is the optical absorption measurements in the UV–visible range which are mainly based on the glass chemical composition. The prepared glass samples had a degree of brownish hue color as shown in Fig. 2 and the absorption spectral shapes were nearly matched with each other before and after gamma irradiation.

**Fig. 3:**
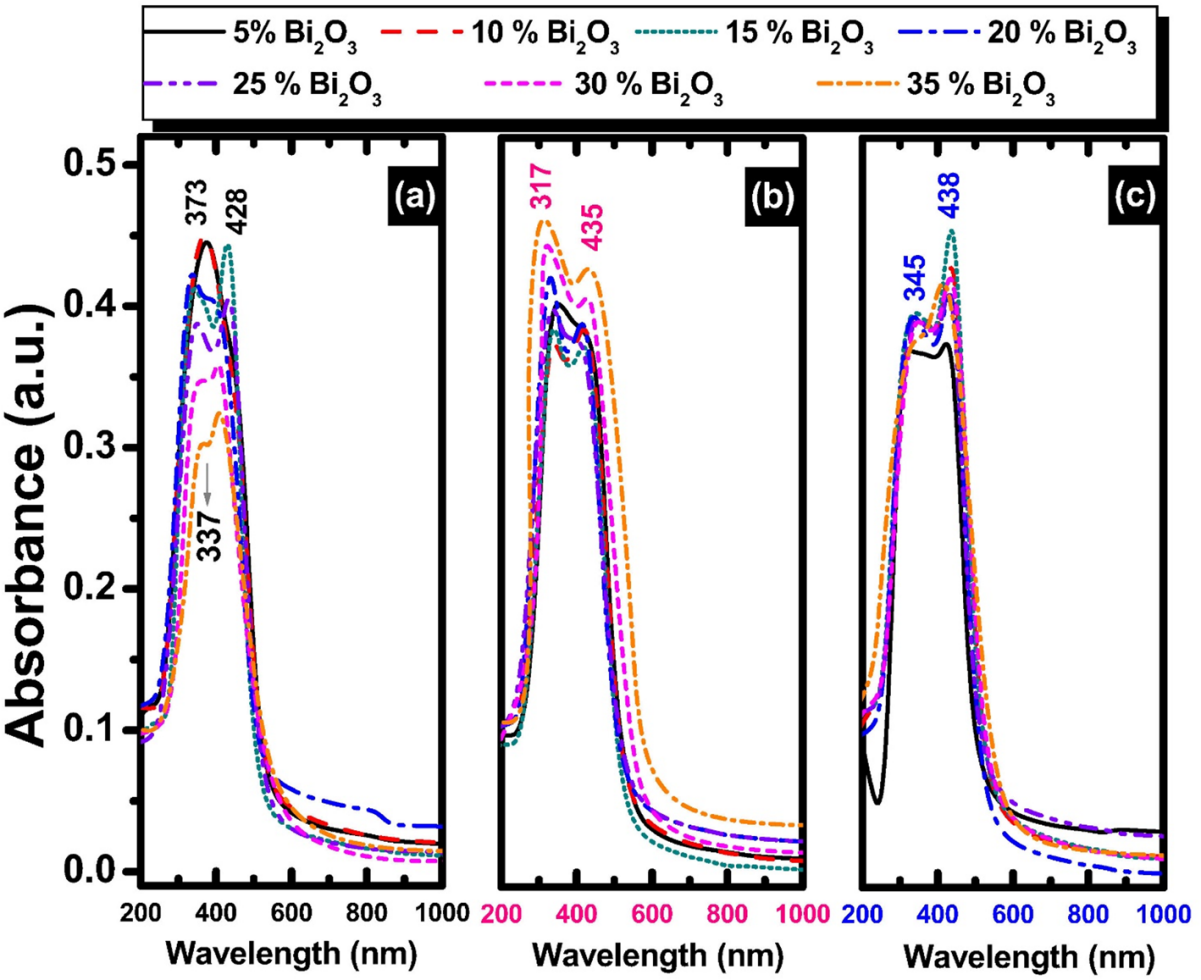
UV–Visible absorption spectra of unconventional binary PbO–Bi_2_O_3_ glasses subjected to different doses of gamma irradiation: (**a**) 0 Mrad (unirradiated), (**b**) 2 Mrad, and **c** 10 Mrad.

Figures 3 shows the absorption spectra of binary non-conventional binary PbO–Bi_2_O_3_ glasses before and after successive 2 or 10 Mrad doses of gamma irradiation. The absorption spectra of glasses before gamma irradiation depict 3 characteristic absorption peaks centered at about 337, 373, and 428 nm. After 2 Mrad gamma irradiation, the observed spectra reveal 2 characteristic absorption peaks centered at about 317 and 435 nm. The glass samples after 10 Mrad gamma dose of irradiation reveal similar absorption spectra that observed after 2 Mrad and the peaks are centered at about 345 and 438 nm. The observed UV and extended visible absorption bands can be attributed to the trivalent bismuth ions Bi^3+^ with 1s^0^ → 3p^1^ transition^28,29^. Many studies^28–33^ have confirmed that the presence of Bi^3+^ is responsible for the appearance of strong UV-near visible absorption spectra. D’Souza et al.^34^ advanced an interesting article concerning the distinct UV absorption peaks identified within Bi_2_O_3_—doped heavy metal borate glass and they confirmed that the shifting of UV absorption to the longer wavelengths was correlated to the additions of Bi_2_O_3_. They revealed the absorption UV peak centered at about 377 nm to the combined effects of Bi^3+^ ions and such peak was suppressed with increasing the content of heavy metal bismuth ions.

The parent binary lead–bismuth glasses show no obvious changes in the UV–visible absorption peaks after successive gamma irradiations 2 & 10 Mrad. Such a result can be attributed to the radiation shielding behavior of chemical constituents of glass that contain heavy metal ions of lead Pb^2+^ and bismuth Bi^3+^ ions that strengthen the glassy network causing shielding for irradiation^35^.

The characterizations of absorption edges are a utile method to detect the band structure in crystalline and non-crystalline materials. The optical transitions that can occur in crystalline or amorphous semiconducting materials are direct or indirect transitions. For both types, the interaction of electromagnetic radiation with electrons in the valence band is responsible for the transitions of these electrons through the fundamental gap to the conduction band.

**Fig. 4:**
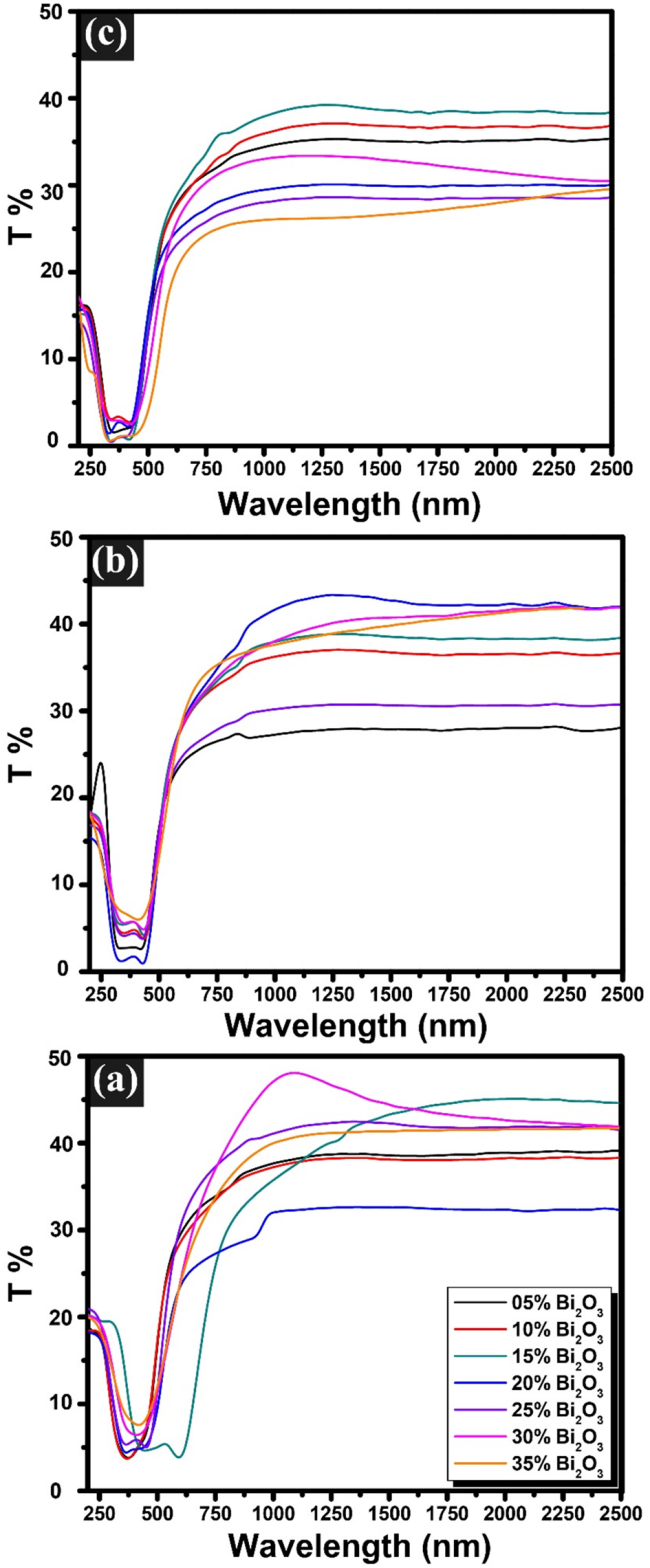
Transmission spectra of unconventional binary PbO–Bi_2_O_3_ glasses subjected to different doses of gamma irradiation: (**a**) 0 Mrad (unirradiated), (**b**) 2 Mrad, and (**c**) 10 Mrad.

The data presented in Fig. 4 illustrates the transmission spectra of the glasses that were prepared for this study. A careful analysis of these results reveals a significant relationship between the optical properties of the glasses and the varying ratios of lead oxide (PbO) to bismuth oxide (Bi_2_O_3_) within the glass composition. As the proportion of these two components changes, it becomes evident that the transmission characteristics of the glasses are notably affected. Furthermore, the investigation also examined the impact of gamma irradiation on the transmission spectra. It was observed that as the dose of gamma irradiation increased, there was a corresponding decrease in the transmission of light through the glasses. This suggests that exposure to higher levels of gamma radiation negatively influences the optical clarity of the glasses, potentially due to structural changes or the formation of defects within the glass matrix.

### Optical bandgap, Urbach energy, and refractive index

**Fig. 5:**
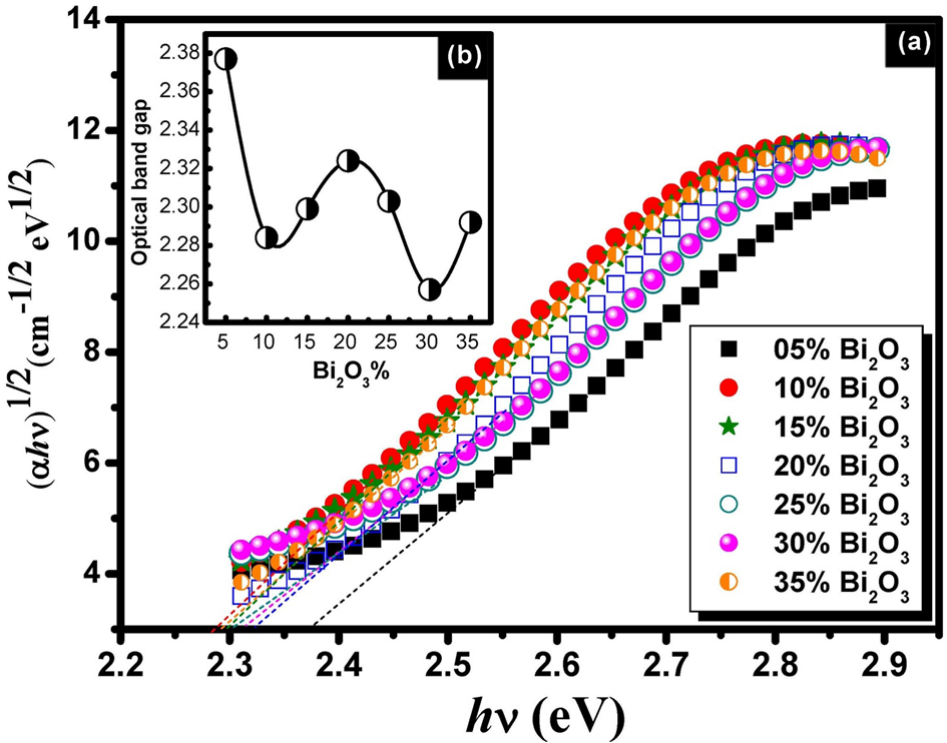
A plot of (αhv)^1/2^ as a function of *hv* for unconventional binary PbO–Bi_2_O_3_ glasses before gamma irradiation (**a**) and the behavior trend of the optical band gap with Bi_2_O_3_ content (**b**).

**Fig. 6:**
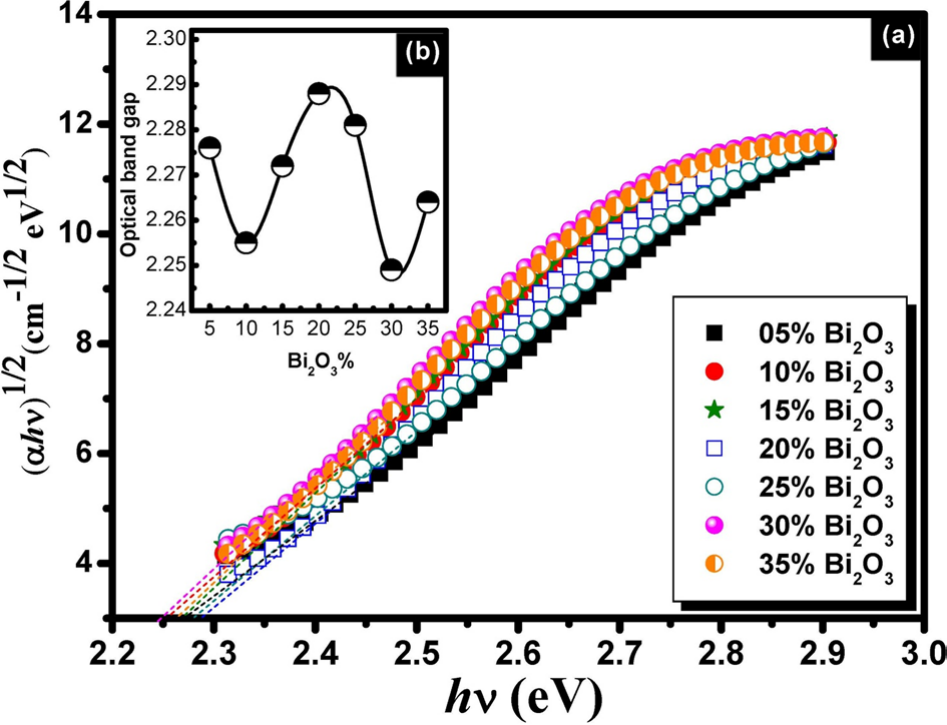
A plot of (αhv)^1/2^ as a function of *hv* for unconventional binary PbO–Bi_2_O_3_ glasses after 2 Mrad gamma irradiation (**a**) and the behavior trend of the optical band gap with Bi_2_O_3_ content (**b**).

**Fig. 7:**
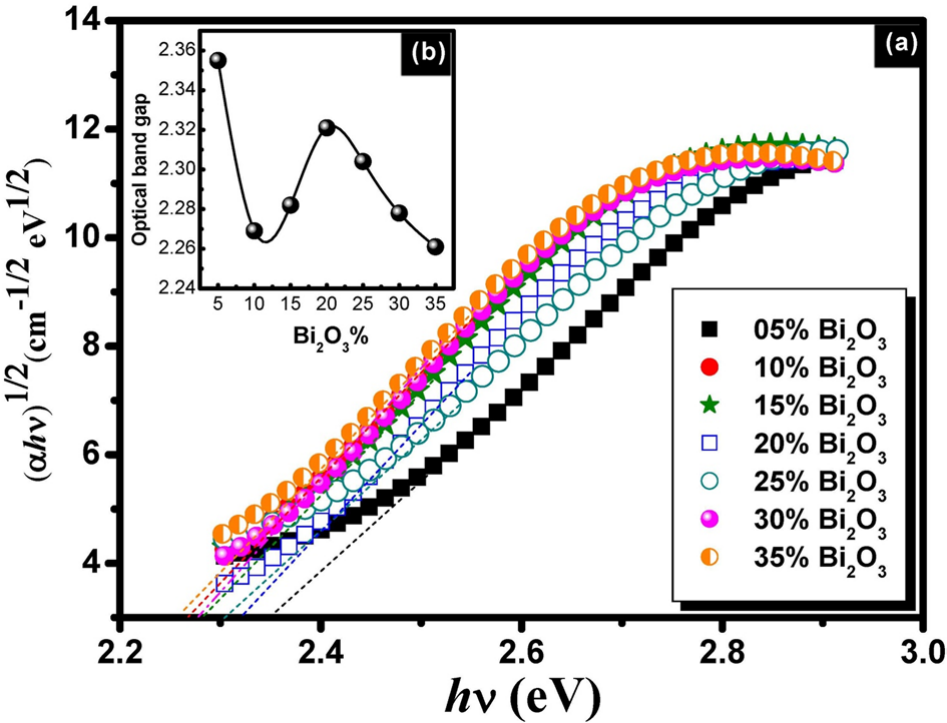
A plot of (αhv)^1/2^ as a function of *hv* for unconventional binary PbO–Bi_2_O_3_ glasses after 10 Mrad gamma irradiation (**a**) and the behavior trend of the optical band gap with Bi_2_O_3_ content (**b**).

Figures 5, 6 and 7 represent the plots of (*αhv*)^1/2^ vs *hv* for binary compositions of non-conventional PbO–Bi_2_O_3_ glasses. The satisfied optical band gap values were obtained by plotting the term (*αhv*)^1/2^ against *hν*. The *E*_*opt*_ was determined from the intercept of the linear portion of this plot with the energy axis.

The plotted curves indicated forbidden indirect transition for the studied glasses. The estimated optical parameters optical band gap, Urbach energy, and refractive index are given in Table 1. The recorded optical parameters were observed to vary with the batch composition of samples. The values of the optical band gap were varied between 2.204 eV as a minimum value and 2.322 eV as a maximum value for glasses before gamma irradiation. On the other hand, a slight decrease and variation were observed after gamma irradiation in the E_opt_ values to be 2.169 to 2.253 eV and 2.175 to 2.285 eV after 2 and 10 Mrad doses of gamma irradiation respectively. Similar variations in the behavior of Urabch energy and refractive index can be observed as shown from the listed values in Table 1. The variation trend in the optical parameters can be attributed to the presence of localized states and dependence on the composition of glasses^36^.

The variation in the optical parameters is correlated to the bond strength and mainly depends on the composition of the glass. The current composition contains two heavy metal oxides PbO and Bi_2_O_3_ with systematic variations in their content. The mentioned oxides play a dual role in glass formations as formers or modifiers according to their content in the glass. The high percent content of lead oxide makes it act as a glass former, while the addition of Bi_2_O_3_ with low content allows the increase of the bond strength by creating non-bridging oxygens which leads to the increase in the optical band gaps^37^. Hence, the glass building structure is mainly correlated to BO or NBO whereas in the current glass model, a part of heavy metal Bi_2_O_3_ may act as a modifying agent that allows the breaking of the bridging oxygen bond to connect with the new cation and the bridging oxygen is deformed into non-bridging oxygen. The introduction of bismuth as modifying cation will decrease the density of BO and increases the NBO content. For instance, the tetrahedral unit with 4BO, Q^4^ will be converted into Q^3^ and then Q^2^ relating to modifying content. Inversely, PbO may allow the formation of NBO when its content becomes lower than Bi_2_O_3_ which is considered as a former in such case. Such variations in the role of heavy metal Pb^+2^ and Bi^3+^ in glass structure interpret the variations of the optical parameters of the studied glasses^36,37^.

On the other hand, Increasing the gamma dose of irradiation will allow an increase in the atomic displacement and the electron density. A large amount of the electrons on the non-bridging oxygen may increase the Urbach tail with an increase in the Urbach energies and gradual decreases in the optical band gap^38^.

A study by Mott and Davis^22^has revealed that in amorphous semiconductors, the optical absorption coefficient depends on the short-range order of the amorphous network matrix. The variation in optical parameters can be interpreted based on the "density-of-states model". The localized states near the mobility edges have a limited width that is affected by the degree of disorder and the induced defects within the amorphous structure. The lowest transmission value observed in the glass can be attributed to the high density of unsaturated bonds, which represent induced defects. These defects increase the number of localized states in the overall structure, leading to higher optical absorption and lower transmission.

According to the previous assumption, the optical estimated optical parametres of the prepared glasses are influenced by its composition, where changes in elemental ratios can lead to shifts in the band structure. Depending on the specific interactions between Bi_2_O_3_ and PbO, the optical band gap could either increase or decrease.

### Photoluminescence measurements

**Fig. 8:**
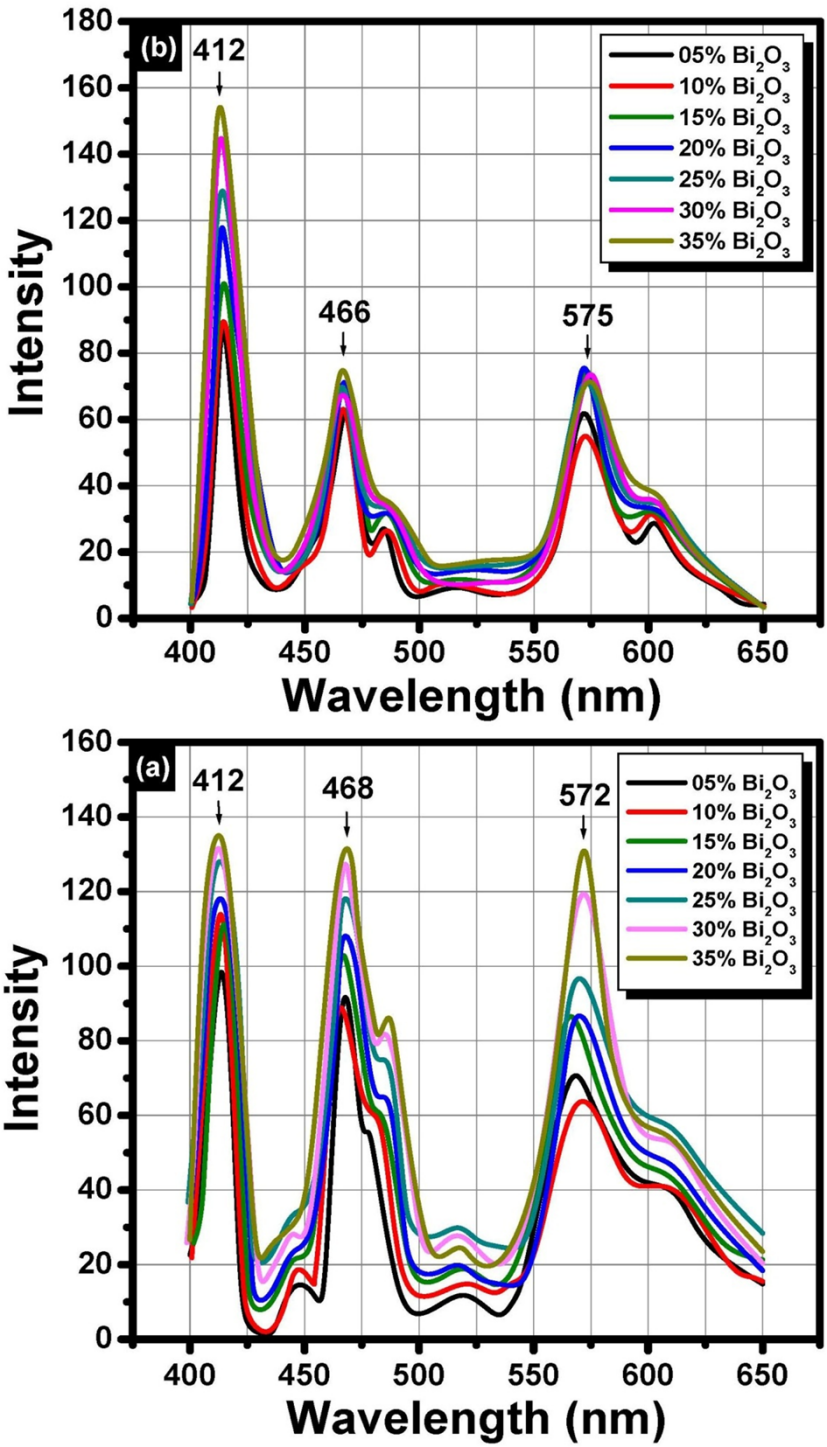
Emission spectra of binary Bi_2_O_3_–PbO glasses at λ_ex_ = 266 nmm (**a**) before irradiation and (**b**) after 10 Mrad gamma irradiation.

**Fig. 9:**
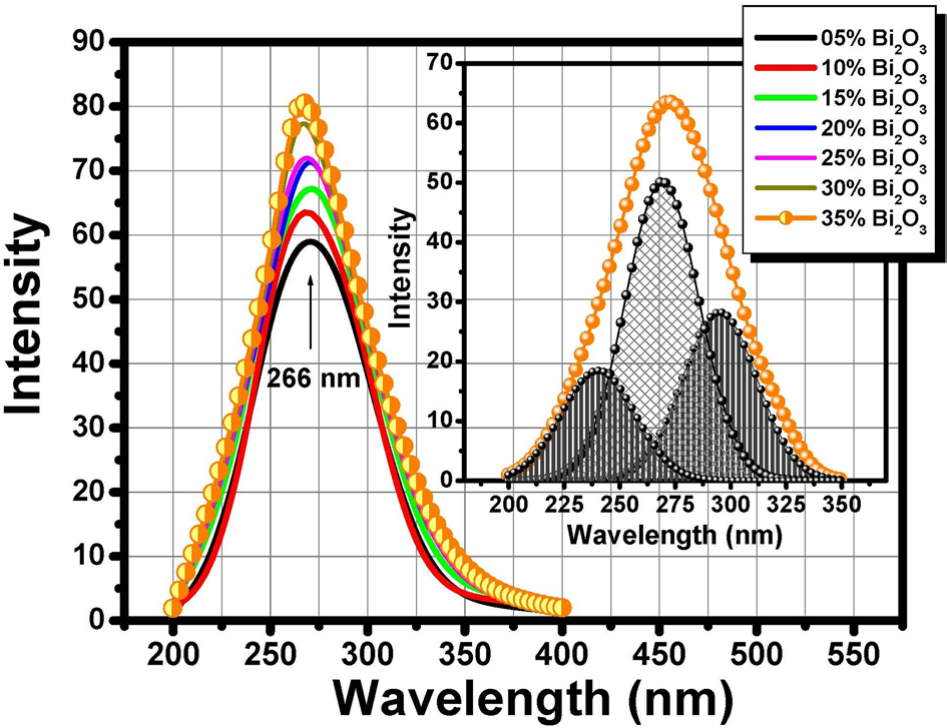
Excitation spectra of binary Bi_2_O_3_–PbO glasses.

The luminescent properties before and after gamma irradiation of the prepared non-conventional binary Bi_2_O_3_–PbO glasses were recorded in the visible range after UV excitation at about 266 nm as illustrated in Fig. 8a and b respectively. According to Fig. 8, the glasses consisted of three main sharp peaks centered at about 412, 468, and 572 nm before gamma irradiation. It can be seen that the emission peaks have the same position with different intensities after 10 Mrad dose of gamma irradiation. The current emission results are matched with that obtained by Denker et al.^39,40^ who assumed that Bi^3+^ is the main source of emission in Mg–Al–silicate glasses under UV excitation at about λ_ex_ = 266 nm. Figure 9 represents the excitation spectral range at emission wavelength 465 nm.

To interpret the PL spectra, Panmand et al.^41^ assumed that the band edge excitation promotes the electron from the valance band to the conduction band by leaving a hole in the valance band then followed by one of 3 processes, the first one is the conduction band electron directly combined with valance band hole with emission of zero photons, then the second is the conduction band electron is trapped by shallow or deep donor like a trap, such trapped electron recombined with a hole with emission of photon process, and finally the conduction band electron re-combined with valance band hole trapped by shallow or deep acceptor like trap and emits the photon.

According to the obtained results, no other valences for bismuth Bi^+^ and Bi^2+^ could be detected and the emission spectra were recorded in the visible region for the current glass compositions with no extended emissions in the Near-IR region that connected to the low-valence bismuth states^39,40^. The interpretation of emission behavior of the non-conventional heavy metal glasses is correlated to the glass’s chemical composition, melting temperature, oxygen pressure, and doping level. The chemical composition of the prepared glasses contains a large content of PbO that has strong absorption in the UV, hence the effect of replacing lead with bismuth was sufficient to cause a shift in both absorption and emission peaks with a characteristic change in the fluorescence intensity^29^. Hence, the Bi^3+^ doped glass is the main activator that shows blue luminescence under excitation by ultraviolet radiation.

Many previous studies^42,43^ indicated that the higher Bi_2_O_3_ content leads to a greater number of Bi^3+^ ions in the glass network. These Bi^3+^ ions can act as sensitizers, absorbing energy and resulting in enhanced emission intensity. Also, Bismuth vacancy-induced enhancement: In some cases, introducing Bi vacancies by reducing the Bi content can significantly enhance the luminescence of bismuth-containing materials. The Bi vacancies create localized energy levels that promote radiative recombination.

**Fig. 10:**
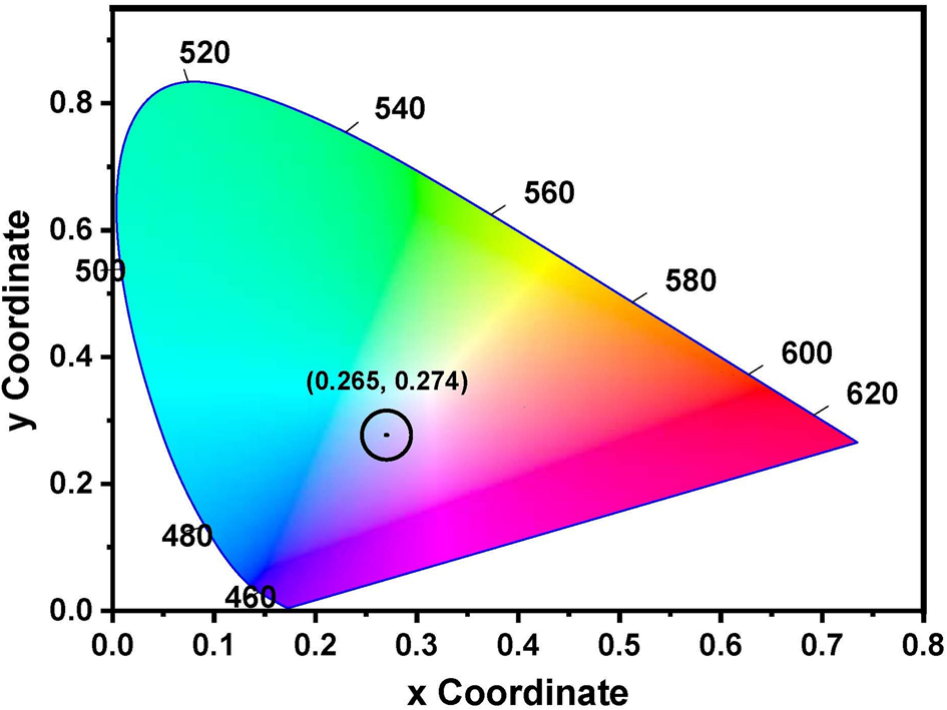
CIE chromaticity diagram of binary Bi_2_O_3_–PbO glasses.

Figure 10 represents the CIE chromaticity diagram of binary non-conventional heavy metal PbO–Bi_2_O_3_ glasses. The color coordinates are located and distributed in the hue violet degree under the UV excitations 266 nm. The actual values of CIE color coordinates were 0.265 and 274. The interpretation of the observed color is due to the ability of lead and bismuth ions to absorb the UV light that emits different degrees of detected violet color emission depending on the type and the ratio of the co-doped compounds^39,40^.

### FTIR absorption measurements

**Fig. 11:**
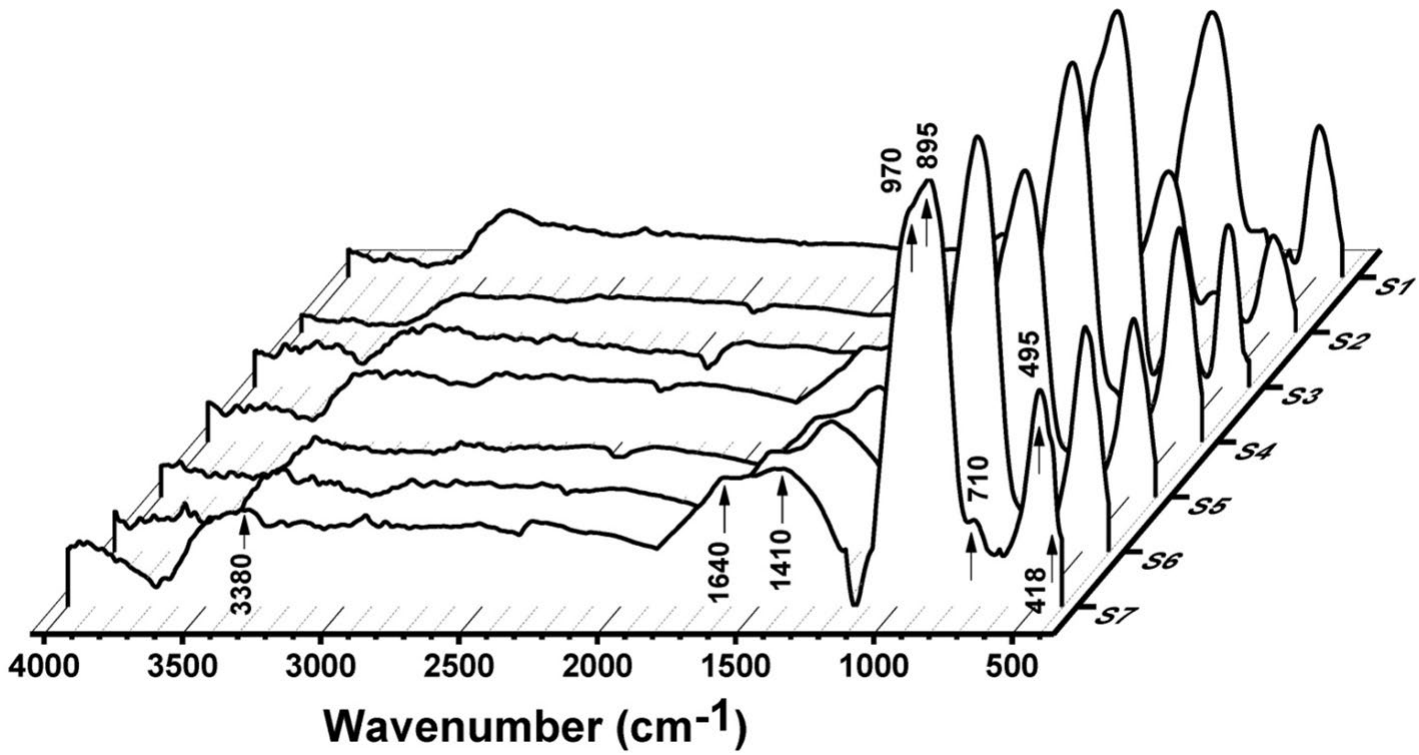
FTIR absorption spectra of the prepared glasses before gamma irradiation.

**Fig. 12:**
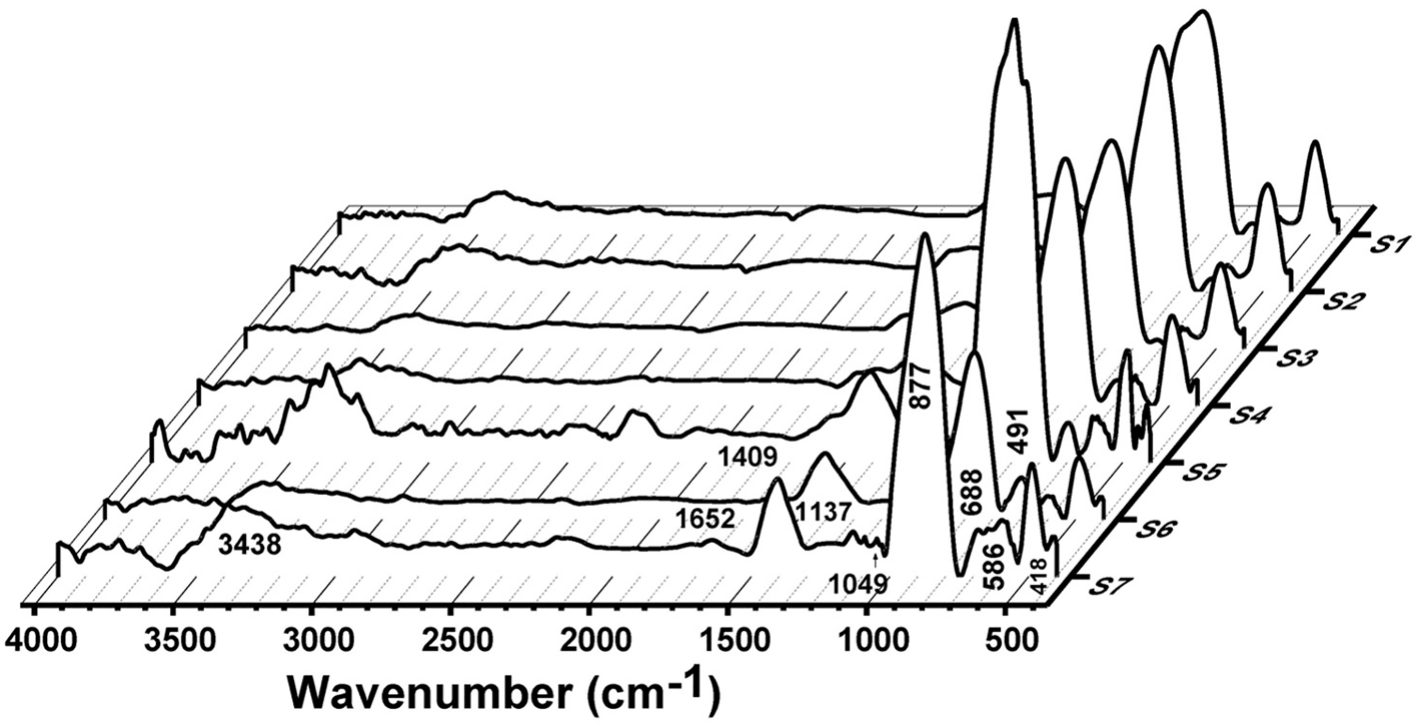
FTIR absorption spectra of the prepared glasses after 10 Mrad gamma irradiation.

The experimental FTIR absorption spectra of all the unconventional binary PbO–Bi_2_O_3_ glass systems are shown in Figs. 11 and 12. The determined bands in the FTIR absorption spectra and their assignments of the current data were compared with those related to glass composition^44–53^. The FTIR absorption spectra before gamma irradiation consist of a shoulder band centered at about 418 cm^−1^ and a sharp intensive peak at about 495 cm^−1^ which can be attributed to the combined bending vibrations of Bi–O from BiO_6_ and/or BiO_3_ and of the Pb–O stretching vibration in PbO_4_ groups^44,45^. Another extended small kink was centered at about 586 cm^−1^ which corresponds to Bi–O from BiO_6_ vibrations^44–47^. Furthermore, a broad shoulder absorption band centered at the range 688–710 cm^−1^ corresponds to the Pb–O bond from the symmetric stretching vibrations of PbO_3_ and or PbO_4_^44^. The spectra show a characteristic sharp high-intensity band at about 895 cm^−1^ with an extended shoulder band centered at about 970 cm^−1^. The sharp band at 895 cm^−1^ is due to the symmetrical stretching vibrations of the Bi–O bond from BiO_6_ octahedral structural units while the shoulder band at 970 cm^−1^ is correlated to the symmetric stretching vibrations of the Pb–O bond arises from different structural modes of PbO_3_ or PbO_4_ or can be attributed to Pb–O–Bi vibrations^44,48^. The low intensive band that is located at about 1049 cm^−1^ is due to the Pb–O asymmetrical bending vibrations^44,49^. The broad wide curvature peak that is centered at about 1409 cm^−1^ can be assigned to O–Pb–O bonds^48,50^. The absorption bands at 1640 and 3380 cm^−1^ can be attributed to the O–H stretching and bending vibration of water due to the absorption of moisture causing the appearance of O–H vibrational modes^48,51^.

The previous FTIR results contribute to understanding the building structure of the current non-conventional binary lead–bismuth oxide as follows^44–53^:

(i) PbO can form stable glass due to the dual role of Pb^2+^ as a network modifier with low concentrations while the formation of Pb–O covalent bond indicated Pb^2+^ as network former exhibiting PbO_3_ and PbO_4_ building units at higher PbO content.
(ii) Bi_2_O_3_ is not a definitive glass former as a result of the prominent polarizability of the trivalent bismuth ion but in the presence of PbO, different ratios of Bi_2_O_3_ can contribute to build a non-conventional glass network.
(iii) The obtained FTIR data illustrate that the glass network is composed of different structural building units from BiO_3_/BiO_6_ and PbO_3_/PbO_4_ depending on the addition ratio between PbO and Bi_2_O_3_.
(iv) The strength of the connectivity between building units of the glass network increases with the increase of Bi_2_O_3_ content.

The FTIR absorption spectra after gamma irradiation reveal no prominent changes in the peak positions, this indicates the general stability of the host lead–bismuth glass towards gamma irradiation due to the presence of a relatively high content of heavy metal oxides that are responsible for keeping electron rearrangement during irradiation^54^.

### Contributions to the effect of bismuth on the optical and structure of the heavy metal-based glass

The optical behavior of bismuth-doped glasses whether it is absorption or emission mainly depends on the building structure of the glass network. For instance, the strong absorption and emissions are increased with the addition of alkali or alkaline earth to bismuth-containing glasses due to their smaller ionic radius that controls the valence states of bismuth ions^54^. It is very important to consider the relationship between the valence state of Bi and the nature of the emitted light of bismuth-containing materials. Murata and Mouri^55^ observed that Bi-doped silicate, borosilicate, borate, and germanate glass has a characteristic special varied IR fluorescence for each glass type while adding Al_2_O_3_ IR fluorescence cannot be obtained and they concluded that the design of glass matrix composition required special conditions to emit the IR fluorescence.

The heavy metal non-conventional Bi_2_O_3_–PbO can’t emit IR fluorescence because Bi exists in its trivalent oxidation state. Previous studies^55,56^ assumed that the origin of NIR emissions is Bi^5+^ and the excepted emissions only occur between the excited levels ^3^D_2_ and ^3^D_3_ and the ground level.

A detailed study by Peng et al.^57^ displayed the interpretation of bismuth photoluminescent behavior that can be concluded as follows;

(i) Estimation of bond energy Bi–O will help to detect the neighboring coordination sphere for Bi-doped glasses. The binding energy increases with the valence state, hence Bi-doped conventional glass formers Silicate, borate, or phosphate prefer the Bi^5+^ valence state^57–62^.
(ii) Type of additions may contribute to remove the stabilization of bismuth NIR fluorescence, for instance, Na_2_O, BaO or Y_2_O_3_ will disappear the IR emission of bismuth containing glasses.
(iii) The IR emission is absent if Bi^5+^ lies in an octahedral environment and has high optical basicity.
(iv) Preparations under a reducing atmosphere or using reducing agents enhance the NIR emissions.

According to the previous assumption the emissions of the prepared glasses don’t obey the previous rule and after exclusion of the presence of Bi^5+^, we suggest that the visible recorded emissions are due to the Bi^3+^ ions. There are no characteristic induced defects after the high gamma irradiations; hence the current composition represents a shielding effect towards gamma irradiation.

## Conclusions

Non-conventional heavy metal binary lead and bismuth oxide from basic chemical composition (100 − x)PbO–xBi_2_O_3_ where x = 35, 30, 25, 20,15,10, and 5 were prepared by the conventional melt and annealing method. Collective spectral analyses were carried out before and after gamma irradiation to evaluate the radiation shielding behavior of the prepared glasses. X-ray diffraction measurement reveals the characteristic amorphous nature of the binary heavy metal-formed glasses. The optical UV–Visible absorption spectra show 3 absorption peaks in the UV region 316, 345, and 380 nm with a characteristic peak in the near-visible region at 430 nm due to the trivalent bismuth ions Bi^3+^ with 1s^0^ → 3p^1^ transition. The optical absorption shows no characteristic changes after 2 and 10 Mrad successive doses of gamma irradiation. The optical band gap varied between 2.204 and 2.322 eV before gamma irradiation while a slight decrease and variation were observed after gamma irradiation. The same variations in the behavior of Urabch energy and refractive index which related to the presence of localized states and dependence on the composition of glasses. Under the UV excitations about 266 nm the glasses consisted of three main sharp emission peaks centered at about 412, 468, and 572 nm before gamma irradiation. The actual values of CIE color coordinates were 0.265 and 274 around the violet color. The FTIR measurements show that the presence of PbO with different ratios of Bi_2_O_3_ can contribute to build a non-conventional glass network. The structural building units of the glass network are formed from collective groups of BiO_6_ and/or BiO_3_ and PbO_4_. According to the structural stability of glass, the method of preparation optimized the glass formation conditions at low temperatures that prevent the volatilization of Bi_2_O_3_ and keep the structure stable compared with other compositions applied for heavy metal oxides. The identified shielding effects of gamma irradiation can be assigned to the chemical constituents of glass that contain heavy metal ions of lead Pb^2+^ and bismuth Bi^3+^ ions that strengthen the glassy network causing shielding for gamma irradiation. The PL behavior and the stability under high doses of gamma irradiations indicated the suitability of the prepared glasses for applications in emission devices and disposal of high-level nuclear waste. Hence, materials based on heavy metal oxides PbO and Bi_2_O_3_ can be used to form effective glass to prevent unnecessary exposure to nuclear radiation.

## Data Availability

The datasets used and/or analyzed during the current study are available from the corresponding author on reasonable request.
